# Structural Insights into Carboxylic Polyester-Degrading Enzymes and Their Functional Depolymerizing Neighbors

**DOI:** 10.3390/ijms22052332

**Published:** 2021-02-26

**Authors:** Ana Lúcia Leitão, Francisco J. Enguita

**Affiliations:** 1 Faculdade de Ciências e Tecnologia and MEtRICs, Campus da Caparica, Universidade Nova de Lisboa, 2829-516 Caparica, Portugal; aldl@fct.unl.pt; 2 Instituto de Medicina Molecular João Lobo Antunes, Faculdade de Medicina, Universidade de Lisboa, Av. Prof. Egas Moniz, 1649-028 Lisbon, Portugal

**Keywords:** polyester, esterase, plastic polymer, biodegradation, depolymerizing esterase, PETase

## Abstract

Esters are organic compounds widely represented in cellular structures and metabolism, originated by the condensation of organic acids and alcohols. Esterification reactions are also used by chemical industries for the production of synthetic plastic polymers. Polyester plastics are an increasing source of environmental pollution due to their intrinsic stability and limited recycling efforts. Bioremediation of polyesters based on the use of specific microbial enzymes is an interesting alternative to the current methods for the valorization of used plastics. Microbial esterases are promising catalysts for the biodegradation of polyesters that can be engineered to improve their biochemical properties. In this work, we analyzed the structure-activity relationships in microbial esterases, with special focus on the recently described plastic-degrading enzymes isolated from marine microorganisms and their structural homologs. Our analysis, based on structure-alignment, molecular docking, coevolution of amino acids and surface electrostatics determined the specific characteristics of some polyester hydrolases that could be related with their efficiency in the degradation of aromatic polyesters, such as phthalates.

## 1. Introduction

Esters are organic molecules originated by the condensation of acids and alcohols. They are widespread in biological systems, participating in metabolism, but also being important components of the cell structure. Considering their relative abundance in cellular structures, carboxylic esters are the most prevalent family of these compounds. Cellular membranes are mainly composed of phospholipids, which are esters of mono-carboxylic fatty acids with glycerol and other alcohols [1]. Multifunctional carboxylic acids are also used by cells to synthesize complex polymeric esters such as plant cutins, which form an impermeable cover for leaves and other plant structures [2].

Mimicking the structure of natural polymeric esters, chemical industry produces several polymers with plastic properties by combining di-carboxylic organic acids with alcohols [3]. Polyesters are the most important family of industrial polymers, being present in all kinds of daily goods including recipients, containers, fabrics and fibers [4]. The chemical nature of the polyester components is related with the physico-chemical and mechanical properties of the polymer. Polyesters synthesized by the esterification of aliphatic alcohols and acids produce low-melting point (50–80 °C) and semicristalline materials with poor mechanical properties. Other polyesters combine aromatic acids with aliphatic alcohols, generating plastics with improved properties, higher melting points (160–280 °C), and useful for applications that involve extrusion and injection methods [5]. Among aliphatic-aromatic polyesters, polyethylene-terephthalate (PET) is one of the most widely used plastics for the fabrication of liquid containers, bottles and machine parts [5,6].

The industrial production of plastics worldwide has increased exponentially in the last years, growing from two million Tons in 1950 to 380 million Tons in 2015 [7]. As stated in the last report of the Ellen MacArthur Foundation, the “plastic economy” has major drawbacks due to the intrinsic stability of these polymers in the environment and the limited extend in their recycling, which can be estimated around 5–10% of the total production. Uncontrolled disposal of PET and other terephthalate-based plastics is an important source of environmental pollution due to their increasing accumulation rates in surface lands and waters in the last decade [7]. Microbes and their intrinsic metabolic capabilities have been described as a potential new strategy for the environmental remediation and valorization of plastic wastes [8].

Strategies for the bioremediation of PET and other polyesters have gained a new impulse since the discovery and isolation of PET-degrading enzymes from marine microorganisms, exemplified by the PETase from *Ideonella sakaiensis* [9]. Polyester-depolymerizing enzymes such PETase are monodomain carboxyl-esterases that belong to the hydrolase family [10]. Carboxyl-esterases are extremely versatile enzymes since they have a relative low substrate specificity and are widespread across all the living kingdoms [11]. They are the result of divergent evolution of monodomain enzymes with an alpha/beta fold and a catalytic center formed by three amino acids: Serine, asparagine and histidine [12]. Carboxyl-esterases catalyze different types of reactions; their primary catalytic activity is hydrolysis, but depending on the substrate concentrations and other reaction conditions they can also catalyze esterification and trans-esterification reactions [11]. The hydrolysis of carboxyl esters is catalyzed by a two-step reaction; first a nucleophilic attack of the catalytic serine will release the corresponding alcohol and an acetylated intermediate of the enzyme, that it is stabilized by the help of the histidine residue in the catalytic center. In a second step, the enzyme will return to its native state by the hydrolysis of the acyl intermediate and the release of the carboxylic acid [13,14].

Carboxyl-esterases maybe classified using different criteria that include their substrate specificity or inhibition patterns. The most complete classification was proposed by Arpigny and Jaeger [15] which distinguish 15 different families of enzymes depending on their substrate specificity, catalytic centers and amino acids involved in catalysis. Taking into account the structural configuration of the catalytic center, we can simplify this classification considering two super-families of carboxyl-esterases: ester hydrolases and lipases. The ester-hydrolase superfamily is composed by alpha/beta monomeric proteins with catalytic activity over mono- and poly-esters, where the active center composed by the catalytic triad Ser-Asp-His is located in a protein cleft exposed to the solvent [11]. In the lipases, the catalytic center is covered by a small domain that limits its accessibility to the substrates and modulates the catalytic activity of these enzymes that are specifically designed to catalyze hydrolysis of ester bonds in hydrophobic environments [10].

Several carboxyl-esterases from bacteria including cutinases, lipases and the specific PETases have been described to degrade plastic synthetic polymers based on phthalate under laboratory conditions [9,16,17]. Even in the case of the specific PETase from *I. sakaiensis*, the extension and yield of the enzymatic degradation is very limited and only possible in amorphous polymers [18]. Highly dense PET polyesters present in bottles and other containers are not able to maintain the growth of bacteria producing plastic-degrading enzymes [19]. These crystalline polymers are also very inefficient substrates for isolated carboxyl-esterases due to their intrinsic hydrophobicity and compactness, that prevent the proper access of the enzymes to the substrate [16,20]. In consequence, the biotechnology applications of plastic-degrading hydrolases would require a deeper knowledge about their mechanism of action and specificity. This knowledge has been recently empowered by the massive metagenomic approaches for the genomic characterization of environmental microbes [9,21], and also by the molecular and structural biology techniques that allowed precise engineering of these enzymes for improving their catalytic properties [22,23].

In this report, we analyzed the structure-activity relationships of different polyester-hydrolases comparing their molecular structures and the surrounding environment of the catalytic center, including their substrate-binding site properties. The obtained results could contribute to the directed engineering of new polyester-degrading enzymes with improved catalytic properties.

## 2. Methods

### 2.1. Structure Comparison

The functional homologs of *I. sakaiensis* PETase (PDB code: 5XJH) were selected by structure comparison with the PDB database using the FATCAT2 algorithm [24]. The structural search was performed by using a non-redundant subset of PDB database containing 51,859 structures and applying a flexible alignment mode. This search was further refined and narrowed to the bacterial carboxyl-esterases by applying the Mustguseal algorithm [25]. The Mustguseal protocol first starts with a structure similarity search to find the relatives of the query structure in different protein families. Each structural relative is then scanned by sequence homology to collect functional homologues, constructing a protein family and a multiple protein alignment [26]. In the structural alignment, the algorithm was applied using X-ray structures from the PDB database and a lowest acceptable match for the target structure of 70%. In the sequence similarity search, Mustguseal software parameters included a redundancy filter threshold of 95% and a dissimilarity filter threshold of 0.5 over the Uniprot/Swiss-Prot databases. The analysis and comparison of enzyme foldings was performed by DALI server [27].

### 2.2. Residue Co-Evolution

The relationships between structure and function, and the identification of hotspots involved in co-evolution patterns in bacterial esterase structures were identified by the visualCMAT algorithm [28]. Structural alignments obtained with Mustguseal were analyzed by visualCMAT, using a Henikoff’s position-based weight and a profile-based adjustment for the determination of co-evolving residues across carboxyl-esterases.

### 2.3. Ligand Docking

Atomic models of polyethylene-terephthalate (PET), polypropylene-terephthalate (PPT) and polybutylene-terephthalate (PBT) were manually built with Maestro software v.12.3 [29]. Hydrogen atoms were automatically added following the riding model and the structures were optimized by 100 cycles of energy minimization. Molecular docking of PET, PPT and PBT models on the surface of *I. sakaiensis* PETase was performed by applying a blind-method with a previous cavity detection and flexible docking using CB-Dock and Autodock Vina [30]. The list of docking solutions was ranked by their internal scores and Gibb’s function.

### 2.4. Data Representation and Analysis

Analysis of protein sequence-structure alignments and construction of the derived unrooted phylogenetic trees was performed by Jalview [31]. The corresponding phylogenetic trees were graphically represented with iTOL v.5 software [32]. Protein structures were analyzed and represented with PyMOL [33] and Protein Imager [34]. Surface electrostatics in PETase and other esterase structures were calculated by the Adaptive Poisson-Boltzman method (APBS) implemented as a plugin within PyMOL [35]. The analysis and comparison of electrostatic properties of ester-hydrolases was performed by the PIPSA algorithm [36] implemented in the web portal webPIPSA [37].

## 3. Results

### 3.1. Carboxyl-Esterase Fold Is Widespread in Prokaryotic and Eukaryotic Proteomes

Polyester-depolymerizing enzymes are carboxyl-esterases, belonging to the superfamily of alpha/beta hydrolases [11]. The structure of these enzymes is very compact, and includes a beta-sheet core formed by eight or nine strands, which is surrounded by alpha helices. The catalytic center is typically constituted by three amino acids, Asp (D), His (H) and Ser (S), which are located in the walls of a deep surface pocket [38]. Ser (S) is generally at the center of the peptapeptide motif GXSXG, where X is any residue [15]. To determine the extension of this catalytic folding across the already characterized structures, we used the atomic coordinates of PETase from *I. sakaiensis* (PDB code: 5XJH) to perform a homology search in an extended subset of non-redundant PDB structures. The results obtained by the application of the FATCAT2 algorithm showed a total of 293 protein structural homologues with *p*-value < 10^−5^ (Supplementary Table S1). The 137 protein structures with *p*-value < 10^−8^ were used to construct an unrooted phylogenetic tree derived from corresponding structure-based sequence alignment (Supplementary Figure S1). Analyzing the structural comparisons, we can conclude that the polyester-hydrolase fold is extended in several enzymes isolated from plants, mammals, bacteria, fungi and archaea with metabolic functions related with the hydrolysis of ester bonds in different biomolecules, including lipids and other natural and synthetic polymers. Interestingly, a significant proportion of alignment hits belong to uncharacterized bacteria samples isolated from metagenomic projects. The results also demonstrate the close structural similarity of PETase from *I. sakaiensis* with bacterial esterases, lipases, cutinases and hydrolases.

**Figure 1:**
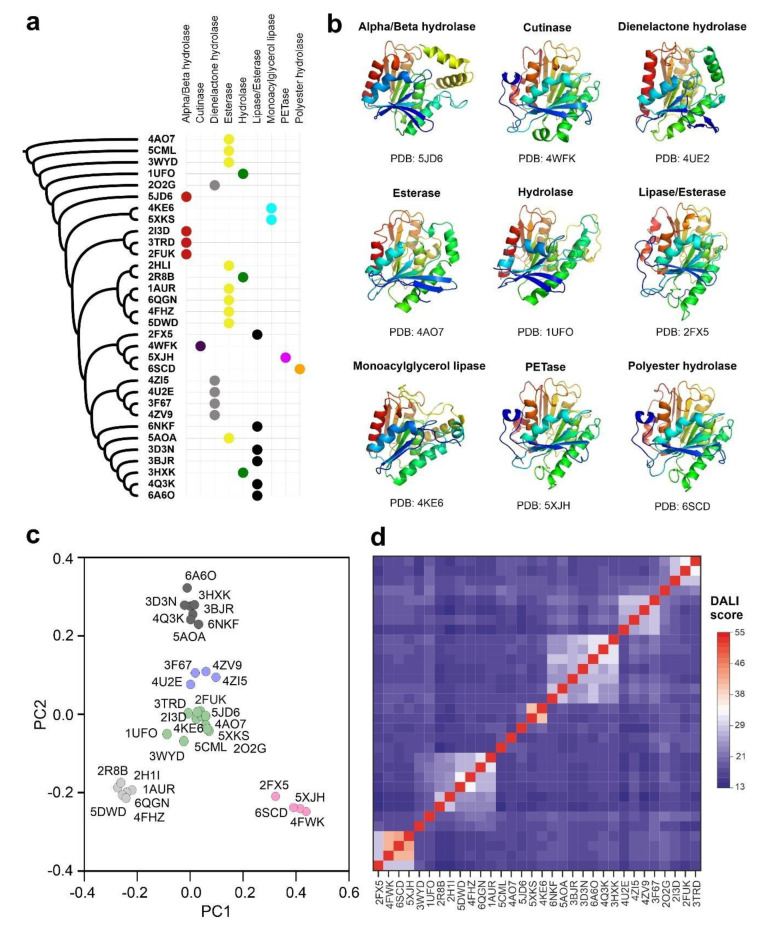
Functional and structural categories of prokaryotic ester-hydrolases showing homology with PETase from *I. sakaiensis*. (**a**) Unrooted phylogenetic tree obtained from the core structural alignment of the 32 bacterial hydrolases with significant homology with the *I. sakaiensis* PTEase (PDB code: 5XJH) by the Mustguseal algorithm, showing the different categories of enzymatic activities represented in the group [26]; (**b**) aligned representative structures for each catalytic group in a ribbon representation and a rainbow gradient coloring (blue to red, from N-terminus to C-terminus); (**c**) principal component analysis of the group of the 32 selected bacterial ester-hydrolases after alignment with DALI algorithm [27]; (**d**) distance matrix of DALI homology scores that clearly depict the clustering of structures with increased folding homology.

Despite the inclusion of a flexible alignment algorithm in FATCAT2 that takes into account the possible structural rearrangements in homologous foldings, the construction of a structure-based sequence alignment has clear drawbacks, especially when the analyzed subset of proteins is very heterogeneous in sequence [39]. The algorithms where the structural alignment is improved by including sequence information are more efficient, reliable and produce better results as stated in recent benchmark studies [40]. To generate a core structural alignment combining sequence and structure information we applied the Mustguseal method [25]. Atomic coordinates of the PETase from *I. sakaiensis* were used as a seeding model to scan non-redundant PDB entries and Uniprot-Swissprot protein sequences simultaneously. We used the structural alignment to build a distance phylogenetic tree and the selected core homologs to perform a statistical analysis of their structure similarities (Figure 1a). The core alignment members harboring the carboxyl-esterase fold could be organized into five main clusters considering the homology score determined by the DALI algorithm and a principal component analysis (Figure 1c,d). The cluster that includes PETase from *I. sakaiensis* also contains three additional members: an alkaline lipase from *Pseudomonas mendocina* (PDB code: 2FX5) [41], a cutinase from *Saccharomonospora viridis* (PDB code: 4WFK) [42], and a polyester hydrolase isolated from *Pseudomonas aestusnigri* (PDB code: 6SCD) [18]. The cutinase from *S. viridis* is a calcium-dependent enzyme with esterase activity and able to depolymerize PET [16]. Calcium ions are essential for inducing structural transitions in the enzyme that are related not only with its catalytic activity but also with its thermal stability [42]. The polyester-hydrolase isolated from the marine bacterium *P. aestusnigri* is structurally close to the *I. sakaiensis* PETase, but it showed decreased performance in the degradation of commercial PET films and containers [18]. The alkaline lipase from *P. mendocina* was employed as one of the components of an enzyme mixture used for the bleaching of lignin and phenolic-derived polymers [43].

### 3.2. Coevolution of Catalytic and Structural Residues in Polyester-Hydrolases

**Figure 2:**
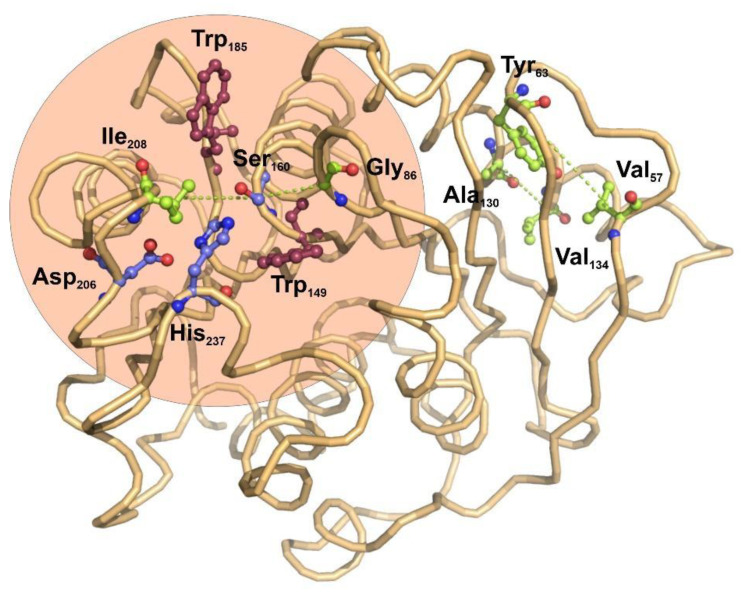
Functional structure and co-evolving residues in bacterial polyester-hydrolases depicted over the structure of PETase from *I. sakaiensis*. The catalytic triad is formed by the central Ser160 and the Asp206 and His237 (blue ball-stick amino acids). The use of visualCMAT algorithm [28] for the study of coevolving amino acid residues across all the bacterial polyester-hydrolases allowed to determine four amino acids pairs that are related by coevolution events (light green ball-stick amino acids and connecting dotted lines). Interestingly, the catalytic center of PETase from *I. sakaiensis* is flanked by two tryptophan residues, Trp159 and Trp185 (magenta ball-stick amino acids), which ensure a local hydrophobic environment. The figure was prepared with Protein Imager [34].

The potential applications of polyester hydrolases in bioremediation and enzyme catalysis would benefit from the detailed knowledge of functional and structural relationships among the amino acid residues found in this family of enzymes. Protein coevolution at the residue level is imposed by structural and functional constraints, and constitutes an important area of study to determine the functional and structural connections among amino acids composing a family of closely-related proteins [44]. To understand the internal residue connections in the selected group of bacterial ester hydrolases, we have performed a residue-based coevolution study by applying the visualCMAT [28] algorithm to their structure alignments. The results of the analysis used the structure of *I. sakaiensis* as a representative structure, serving as a scaffold for depicting the relationships among amino acids (Figure 2).

The score ranking of all the coevolving positions clearly showed that Ser160, the main catalytic residue of esterases, has the higher tendency to participate in coevolving relationships with other amino acids (Supplementary Tables S1 and S2). Interestingly, in the core alignment of 32 bacterial carboxyl-esterases, catalytic Ser160 establishes coevolution relationships with two hydrophobic residues located in the molecular boundaries of the catalytic pocket, Ile208 and Gly86 (Figure 2). These results are compatible with the previous observations indicating that site directed mutagenesis of the amino acids located in the boundaries of the active center have an important effect on the catalytic activity of *I. sakaiensis* PETase over small organic esters [45]. There is no evidence for coevolution involving the remaining elements of the catalytic triad, Asp206 and His237.

Far from the catalytic center, two other pairs of coevolving residues were determined: Tyr63-Val57 and Ala130-Val134. Both pairs of hydrophobic residues are located in the interface between a beta-strand and an alpha helix, opposite to the active center.

### 3.3. Substrate Binding and Surface Electrostatics

The hydrolase alpha/beta fold is widespread in nature and produces enzymes that have an extended range of catalytic activities and substrates. However, the specificity and catalytic efficiency of some microbial esterases over plastic polymers is still an intriguing observation. The structural and functional elements present in microbial plastic-hydrolases such as PETases or polyester hydrolases that are responsible for their catalytic specificity, remain largely uncharacterized. Previous studies, based on protein sequence homology and site directed mutagenesis, showed that the catalytic activity of PETases is influenced by two subdomains involved in substrate binding and protein compactness [20,46]. These subdomains are also useful to classify the different bacterial PETases in three families: type I, type IIa and type IIb [46]. Type II enzymes are more efficient in the degradation of PET and other synthetic polyesters, and have two distinct features that distinguish them from the type I PETases: the presence of an additional disulfide bridge between cysteines 203 and 239, and the existence of an extended amino acid loop in the C-terminal region of the protein comprising residues 242 to 247 [46].

**Figure 3:**
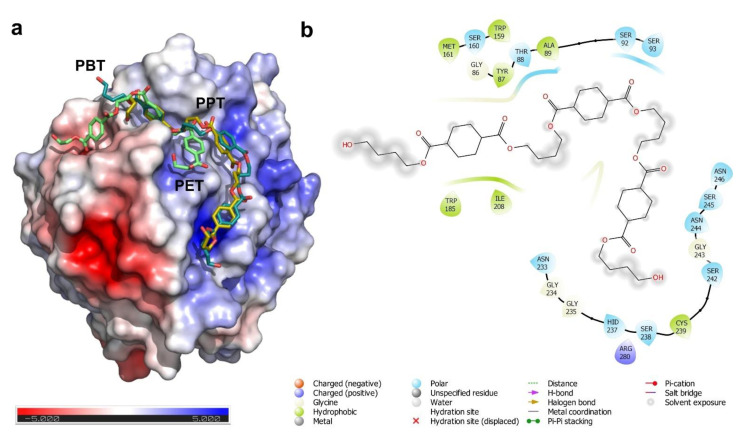
Electrostatics and binding of synthetic polyesters in the surface of PETase from *I. sakaiensis*. (**a**) The results of the molecular docking analysis of three synthetic polyesters (PET, PPT and PBT) onto the surface of PETase protein. The molecular surface is colored by its electrostatic potential as calculated by the APBS algorithm, using the color ramp scale depicted at the bottom of the panel. The electrostatic surface demonstrates the presence of a positive-charged patch of amino acids that could help to the interaction of the aromatic monomers of the polyesters. (**b**) Interaction map showing the amino acids involved in the binding of PETase with PBT and all the putative chemical interactions. The protein surface amino acids involved in this interaction are mainly hydrophobic and with a residual positive charge (Asn233, His237, Asn 244, Asn246 and Arg280). This surface charge distribution forms a positive-charged hinge that will favor the cation-pi interactions with the aromatic residues present in the phthalate-based plastic polymers.

The analysis of the surface electrostatics of *I. sakaiensis* PETase by the adaptive Poisson-Boltzman method showed the presence of an asymmetrical pattern of surface charge distribution that divides the protein into two halves with opposite electrostatic charges, being the catalytic cleft in the border of both regions (Figure 3a). We hypothesized regarding the possible implications of this charge distribution in the substrate binding mechanism of the enzyme, its catalytic efficiency and substrate specificity. To study the surface patches involved in substrate binding, we performed molecular docking experiments using three different models of synthetic polyesters: Polyethylene-terephthalate (PET), polypropylene-terephthalate (PPT) and polybutylene-terephthalate (PBT). For the docking experiments we used short polymeric structures composed by three monomers in each case, applying a flexible docking protocol for the ligand and the protein using the CB-Dock algorithm [30]. Together with the inclusion of a flexible atomic model, the CB-Dock algorithm has the additional advantage of being a totally blind method by which the protein surface is firstly scanned for the presence of cavities where the ligand will be docked.

The results of the docking analysis depicted in Figure 3 are in agreement with previous molecular modelling studies for the interaction of PETase with PET polymer [20,47]. All the polyester models are predicted to interact with the catalytic cleft, formed by the Ser160, Met161 and the conserved Trp185. This last residue is conserved in all PETase families and ensures a hydrophobic interaction platform for their substrates. Interestingly, polymers formed with propyl and butyl spacers (PPT and PBT) appear to have slightly different binding properties. The presence of three and four carbon atoms between the phthalate groups extended the contact of the polymer with the surface of the protein (Figure 3a). These extended molecular contacts for PPT and PBT are based on cation-pi interactions between the aromatic ring of the phthalate group of the plastic monomers and the positive charged protein surface residues (Asn233, His237, Asn244, Asn246 and Arg280) (Figure 3b).

**Figure 4:**
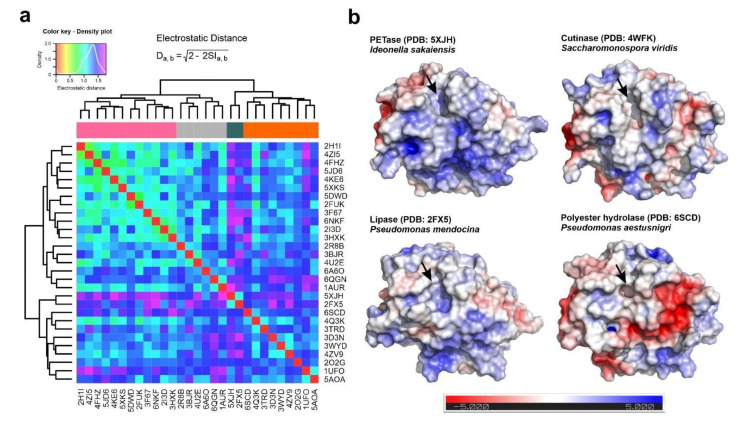
Electrostatic comparison of the structural homologs of PETases and individual analysis of electrostatic surfaces in selected examples. (**a**) Hierarchical clustering analysis of the electrostatic distance matrix for the selected group of carboxylic ester hydrolases structures calculated by the webPIPSA algorithm and the Poisson-Boltzman equation [37]. The electrostatic distances between protein pairs are represented by the rainbow color scale depicted in the figure legend, and the proteins are identified by their PDB codes. (**b**) Electrostatic surface representations of representative carboxyl-esterases harboring experimental evidence of their plastic-degrading capabilities. The molecular surface is colored by its electrostatic potential as calculated by the APBS algorithm implemented in the corresponding PyMOL plugin [33], using the color ramp scale depicted at the bottom of the panel. The position of the pocket that contains the catalytic triad Ser-His-Asp is represented by an arrow.

The detailed analysis of the docking and electrostatics results on PETase from *I. sakaiensis* opened several questions related to the degree of conservation of this electrostatic surface pattern in other polyester hydrolases and the inferred structure activity relationships. To compare the electrostatic surface distribution in other polyester hydrolases, we selected the previously set of bacterial ester hydrolases and analyzed it by the PIPSA algorithm [36,37]. The global electrostatic potential in every structure was compared by the calculation of the individual electrostatic distances, and the numeric matrix used for the construction of a heatmap (Figure 4a). The hierarchical clustering analysis of the data showed the presence of four different clusters of enzymes according to their electrostatic surface profiles, where the *I. sakaiensis* PETase (PDB code: 5XJH) appeared to be closely related only with the *P. mendocina* lipase (PDB code: 2FX5). In a closed view, the electrostatic potential over the PETase surface resembles those observed in the cutinase from *S. viridis* (PDB code: 4WFK) and the lipase from *P. mendocina*. Interestingly, the surface charge distribution differs from the observed in another polyester hydrolase isolated from the marine bacterium *P. aestusnigri* (PDB code: 6SCD), that shows a negatively charged patch close to the catalytic cleft (Figure 4b). The polyester hydrolase from *P. aestusnigri* was already characterized as a PET-degrading enzyme, but its catalytic efficiency exclusively demonstrated the depolymerization of amorphous PET, being unable to degrade high-density variants of the polymer like those used for the production of plastic bottles [18].

## 4. Discussion

Plastic polyesters are widely used polymers for the manufacturing of daily objects and their accumulation in the environment is a relevant problem. The currently implemented valorization measures are clearly insufficient to give a timely answer to plastic pollution in earth and aquatic environments. In this context, the importance of plastic bioremediation processes by microorganisms or microbial enzymes has been increased in the last decade [48]. The first evidence for the microbial degradation of polyester plastics such as PET was described in *Thermobifida fusca*, a thermophilic bacterium isolated from decaying organic matter [49]. The enzyme responsible for this degrading activity was characterized as a serine hydrolase, functionally related to triacylglycerol lipases [50]. In the following years, several microorganisms and enzymes able to degrade PET and other polyesters were described [42,47,51]. The most well studied system is the PETase isolated from the marine bacterium *I. sakaiensis*, which showed an increased degradation yield when compared with other similar enzymes [20]. However, the number and diversity of polyester-degrading enzymes is expected to be expanded in the following years with the onset of the high-throughput metagenomic analysis of natural samples [21,52].

PETases and other plastic-degrading enzymes belong to the serine-hydrolase family, a widely distributed group of catalysts characterized by their relatively low substrate specificity and plethora or different activities [11,13]. Despite this catalytic diversity, their molecular structure is strongly conserved and, in many cases, shows no clear features that could classify a particular protein into a specific catalytic group. In this work, we performed a structure-based analysis of polyester hydrolases using the PETase from *I. sakaiensis* as a reference, with the objective of finding structural features that could be related with the protein function. The generated knowledge could be applied, either for the screening and discovery of new members of the protein family or for the engineering and improvement of already known enzymes.

Our data generated by structure-based search, concluded that the alpha/beta fold observed in PETase is distributed across different proteomes. However, this particular folding is more associated with hydrolytic enzymes that act over branched polyesters in the form of high-molecular weight polymers. This group includes the bacterial cutinases and lipases, together with the already characterized polyester-hydrolases [53]. Among them, the lipase from *P. mendocina* has a particular interest, since it has been already characterized as active over polymeric complexes such as lignin for enzymatic bleaching [43]. This bacterial alkaline lipase has an unusual structure, without the characteristic lid domain that covers the catalytic center [23]. Other interesting structural homologs are represented by the family of cutinases, where the thermostable calcium-dependent cutinase from *S. viridis* is the most relevant example. Despite its non-canonical mechanism and structure, the PET hydrolytic activity of this enzyme has been demonstrated over a wide range of low-density PET polymers [16,42]. Comparing the structural homologs and their enzymatic activity we can conclude that polyester hydrolases active over synthetic PET and other polymers would require the presence of an open active center with direct contact to the solvent [10]. These results justify the low PET degradation efficiencies observed in canonical lipases where the active center is not directly accessible to the solvent. However, recent evidences showed that this catalytic limitation for lipases in PET degradation could be circumvented by synergistic associations with other enzymes, exemplified by a natural consortium formed by five species of *Bacillus* and *Pseudomonas* which is able to growth on PET as a sole source of carbon [54].

PET-degrading enzymes are the result of a divergent evolution of hydrolases that, due to their relative substrate promiscuity, have been specialized to degrade very different ester polymers [12]. At this point, it is difficult to state if the existence of specific PET-degrading enzymes, as the one isolated from *I. sakaiensis*, is a result of environmental pressure or just an adaptation to a wide range of substrates. To infer the importance of amino acid coevolution in this group of enzymes, we performed an analysis with a selected group of PETase structural homologs. The problems derived from covariance models when protein sequences are analyzed, were minimized by the application of the visualCMAT algorithm that detectcs coevolution pairs in structure-based sequence alignments [28]. Our results showed that with the exception of the catalytic Serine residue, the remaining coevolution pairs among the analyzed structures were amino acids located within structural elements, and located at distances not compatible with a molecular interaction. This fact is in agreement with the previous observations obtained in a large cohort of protein domains extracted from the PFAM database [55]. Using the *I. sakaiensis* PETase as representative structure, two coevolving residue pairs Ser160-Gly86 and Ser160-Ile208 were located in the boundaries of the catalytic pocket. Both coevolutionary pairs constitute a network which is spatially close and statistically coupled, suggesting an important role in the structure and function of the esterase center [56]. However, no coevolution signal was detected between the residues involved in the catalytic triad, Ser160, Asp206 and His237. Since this triad is directly involved in the protein function and any variance could modify the catalytic properties of the enzyme, this constitutes a constraint that does not allow any variation and thus does not allow any covariation [57]. Interestingly the remaining coevolving pairs Val57-Tyr63 and Ala130-Val134 are located in a region opposite to the catalytic center and involved in the structural alpha-beta packing of the enzyme. These coevolution pattern suggest a structural constraint in the alpha/beta fold that stabilizes the protein core without modifying the catalytic center, as already has been observed in other proteins such as synthetases or transcription factors [57,58].

However, it is still not clear what are the structural features that make PETase from *I. sakaiensis* so efficient and unique in the PET degradation when compared with other similar enzymes with polyester hydrolase activity. These features are not related to the amino acids involved in enzyme activity or with the protein folding, since they are well conserved in other esterases [11,13]. We postulated regarding the involvement of the surface electrostatics in the interaction with polymeric substrates and in the overall catalytic yield. The comparison of the electrostatic potential function at the surface of the selected group of carboxyl esterases showed that PETase has a unique profile, measured as the electrostatic distance with other enzymes. This profile is partially shared with the bacterial cutinase from *S. viridis* and the lipase-like enzyme from *P. mendocina*. Combining the electrostatic information with the docking experiments, we clearly connected the presence of a positively-charged hinge in the surface of PETase structure (Asn233, His237, Asn 244, Asn246 and Arg280 residues) with the putative substrate-binding site (Figure 3 and Figure 4). Based on an analysis of the results, we proposed that the increased efficiency of *I. sakaiensis* in the degradation of PET and other polymers is related with the enhanced affinity of the substrate for the protein surface. The substrate-enzyme interaction is mediated by the establishment of a stretch of cation-pi interactions between the aromatic rings of phthalate groups and the positive charged hinge along the protein surface. The positively charged surface hinge is absent in less efficient enzymes, such as the polyester hydrolase from *P. aestusnigri*, and only partially present in the *P. mendocina* lipase and *S. viridis* cutinase. In the *P. aestusnigri* polyester hydrolase, experimental evidence showed the inefficiency of the enzyme to depolymerize high-density PET, and the limited activity over the amorphous polymer [18]. The absence of the positively charged hinge in this enzyme suggests a limited substrate interaction when compared with *I. sakaiensis* PETase, which is more evident in the case of crystalline PET.

Since the discovery of PET hydrolases, protein engineering has been applied to the improvement of their catalytic properties. Structural biology data combined with computer methods for protein design have been mainly focused in the improvement of protein stability. Son and coworkers applied a rational protein engineering method based on sequence homology to design a *I. sakaiensis* PETase mutant (S121E-D186H-S242T-N246D) that showed prolonged enzymatic activity over 20 days [59]. Moreover, Cui and coworkers developed a computational strategy for the mutational analysis of PETase and the design of a multiple mutant with enhanced thermostability when compared with the wild-type protein. This mutant designated as “duraPETase” contains the substitutions S214H-I168R-W159HS188Q-R280A-A180I-G165A-Q119Y-L117F-T140D, and it was able to degrade amorphous PET, but also longer polymers such as PBT with an optimum reaction temperature of 40 °C [60]. However, both engineered PETase mutants showed low efficiency in the degradation of crystalline high-density polymers, suggesting that the compactness of the material limits the protein access to the free chemical groups. To solve this problem, some authors have proposed the use of enzyme cocktails. The rate of PETase hydrolysis could be significantly increased in the presence of proteins such as hydrophobin RolA [61]. Hydrophobins represent a class of small fungal protein that has a high surface-active substance and can spontaneously self-assemble at hydrophilic-hydrophobic interfaces, increasing the exposure interface [61].

Our results suggest that the interaction between the enzyme and the substrate could be improved by rational design of the binding interface, since the polyester hydrolases showing a higher PET degradation efficiency contain an interaction surface hinge with a positive charge (Figure 4). This strategy could be useful for the degradation of high-density polyesters harboring aromatic residues, but also can open a new avenue for de novo design of more efficient depolymerases, based on the structural chassis of bacterial PETases and lipases.

## Data Availability

The analyzed data within the manuscript was extracted from PDB (Protein data bank) archive. (https://www.rcsb.org/) (accessed on 26 February 2021).

## Supplementary Materials

The following are available online at https://www.mdpi.com/1422-0067/22/5/2332/s1, Figure S1: Unrooted phylogenetic tree obtained from the structure-based alignment of carboxylic-ester-hydrolases and their structural neighbors using the FATCAT 2.0 algorithm, Figure S2: Secondary structure cartoons of selected plastic-degrading bacterial carboxyl esterases represented by Pro-origami, Table S1: Output from visualCMAT coevolution analysis, including the list of predicted correlated aminoacid pairs and their scores, considering the representative structure of the group (PETase from I. sakaiensis, PDB code: 5XJH), Supplementary Table S2: Output from visualCMAT coevolution analysis, showing the sum of predicted correlation scores per each aminoacid considering the representative structure of the group (PETase from I. sakaiensis, PDB code: 5XJH).
