# Supplementary material for: Structural Insights into Carboxylic Polyester-Degrading Enzymes and Their Functional Depolymerizing Neighbors
Source: Int J Mol Sci. 2021 Feb 26;22(5):2332. doi: 10.3390/ijms22052332 (PMC7956259; doi:10.3390/ijms22052332)
Supplement: Supplementary file 1 [file ijms-22-02332-s001.pdf]

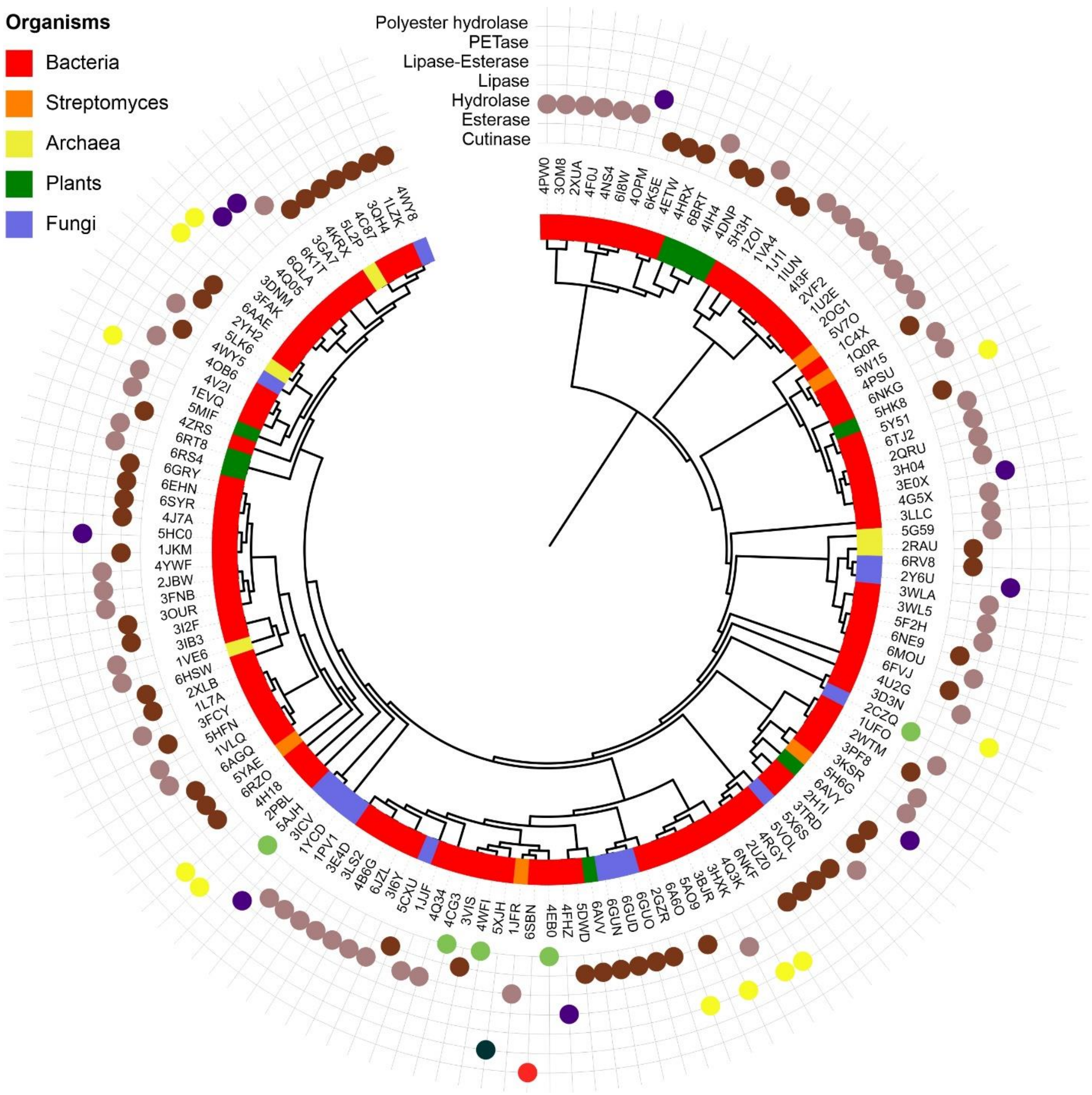

Supplementary Figure S1. Unrooted phylogenetic tree obtained from the structure-based alignment of carboxylic-ester-hydrolases and their structural neighbors using the FATCAT 2.0 algorithm [1]. The phylogenetic tree was constructed and graphically depicted with the iTOL software. Enzymes are represented by their PDB codes, annotated catalytic activities (outer circle) and producing organisms (inner circle) [2].

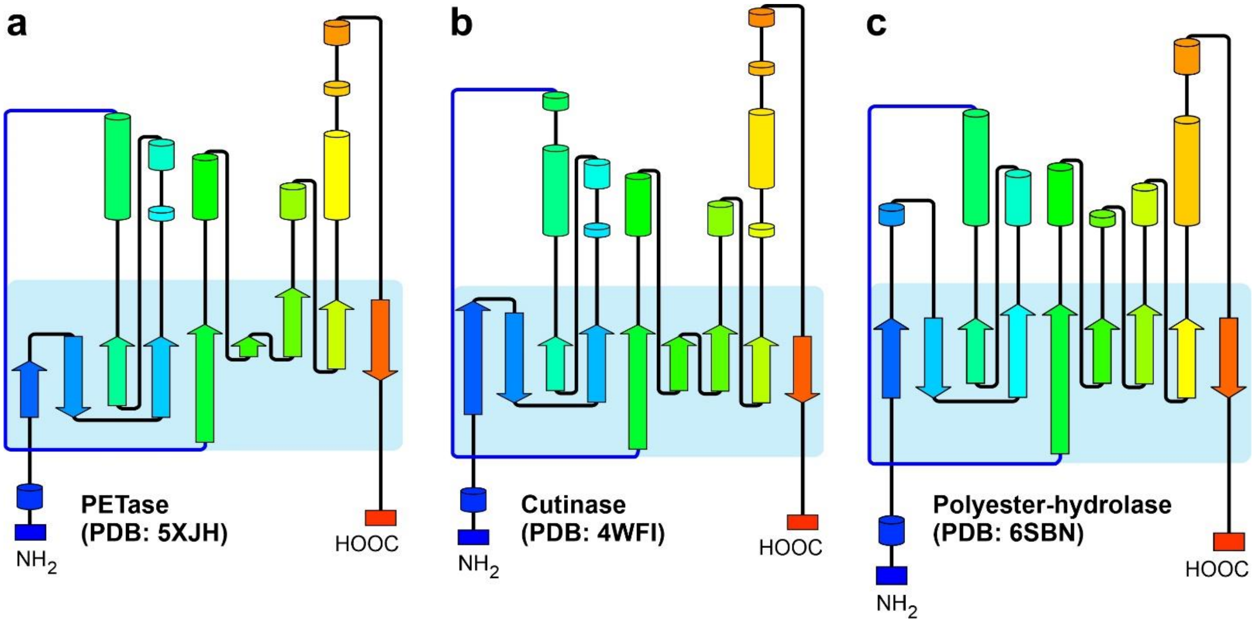

Supplementary Figure S2. Secondary structure cartoons of selected plastic-degrading bacterial carboxyl esterases represented by Pro-origami [3]. The alpha-beta fold is composed by nine central strands surrounded by alpha helices. PETase from *I. sakaiensis* (PDB code: 5XJH) and cutinase from *S. viridis* (PDB code: 4WFI) have a similar distribution of secondary structure elements. In the polyester-hydrolase from *P. aestusnigri* (PDB code: 6SBN) the N-terminal domain contains an additional short alpha helix segment between the first two beta strands, which increases the flexibility of this region.

Supplementary Table S1: Output from visualCMAT coevolution analysis [4], including the list of predicted correlated aminoacid pairs and their scores, considering the representative structure of the group (PETase from *I. sakaiensis*, PDB code: 5XJH). The list of predicted correlated pairs is ranked in decreasing order of the Z-scores; larger Z-scores indicate more statistically significant correlations [5,6]. For each position its ID in the representative protein structure (PDB-ID) and its ID in the sequence (Seq-ID) which corresponds to that representative protein in the multiple sequence alignment, are provided. PDB_id - position`s ID in the PDB file; SEQ_id - position`s ID in the sequence; MIp, MIc, Zp, Zc - the values of the MI-based statistics and the corresponding statistical significance Z-scores.

Rank	*Seq-ID-1*	*PDB-ID-1*	*Seq-ID-2*	*PDB-ID-2*	*MIp*	*Mic*	*Zp*	*Zc*
1	G53	GLY86	S127	SER160	0.332	0.267	7.268	4.793
2	A97	ALA130	V101	VAL134	0.353	0.219	7.7712	4.054
3	V24	VAL57	Y30	TYR63	0.237	0.203	5.181	3.806
4	V24	VAL57	G29	GLY62	0.213	0.189	4.650	3.597
5	S127	SER160	I174	ILE208	0.246	0.187	5.386	3.562

Supplementary Table S2: Output from visualCMAT coevolution analysis [4], showing the sum of predicted correlation scores per each aminoacid considering the representative structure of the group (PETase from *I. sakaiensis*, PDB code: 5XJH). For each position its ID in the representative protein structure (PDB-ID) and its ID in the sequence (Seq-ID) which corresponds to that representative protein in the multiple sequence alignment, are provided [5]. Sum-of-Z-scores are calculated for each position i over all its predicted

correlations with other positions j, but only for those pairs (i, j) that have passed the structural filtering imposed by protein tridimensional alignment. A Sum-of-Z-scores=0 for a position i means that this position was predicted as correlating with other positions j in the multiple sequence alignment but all corresponding pairs (i, j) did not pass the structural filtration. Larger Sum-of-Z-scores indicates a position which tends to participate in a larger number of correlations or in stronger correlations with other positions.

PDB-ID	***Seq-ID***	***Sum of Z scores***
SER160	S127	8.355
GLY86	G53	4.793
ALA130	A97	4.054
VAL134	V101	4.054
VAL57	V24	3.806
TYR63	Y30	3.806
ILE208	I174	3.562
GLY62	G29	0

Additional references

1. Li, Z.; Jaroszewski, L.; Iyer, M.; Sedova, M.; Godzik, A. FATCAT 2.0: towards a better understanding of the structural diversity of proteins. *Nucleic Acids Res* **2020**, *48*, W60–W64, doi:10.1093/nar/gkaa443.
2. Letunic, I.; Bork, P. Interactive Tree Of Life (iTOL) v4: recent updates and new developments. *Nucleic Acids Res* **2019**, *47*, W256–W259, doi:10.1093/nar/gkz239.
3. Stivala, A.; Wybrow, M.; Wirth, A.; Whisstock, J.C.; Stuckey, P.J. Automatic generation of protein structure cartoons with Pro-origami. *Bioinformatics* **2011**, *27*, 3315–3316, doi:10.1093/bioinformatics/btr575.
4. Suplatov, D.; Sharapova, Y.; Timonina, D.; Kopylov, K.; Svedas, V. The visualCMAT: A web-server to select and interpret correlated mutations/co-evolving residues in protein families. *J Bioinform Comput Biol* **2018**, *16*, 1840005, doi:10.1142/S021972001840005X.
5. Suplatov, D.; Sharapova, Y.; Svedas, V. Mustguseal and Sister Web-Methods: A Practical Guide to Bioinformatic Analysis of Protein Superfamilies. *Methods Mol Biol* **2021**, *2231*, 179–200, doi:10.1007/978-1-0716-1036-7_12.
6. Suplatov, D.A.; Kopylov, K.E.; Popova, N.N.; Voevodin, V.V.; Svedas, V.K. Mustguseal: a server for multiple structure-guided sequence alignment of protein families. *Bioinformatics* **2018**, *34*, 1583–1585, doi:10.1093/bioinformatics/btx831.
